# Contraceptive Counselling and Uptake Among Female Kidney Transplant Recipients in Ethiopia

**DOI:** 10.1002/puh2.215

**Published:** 2024-07-08

**Authors:** Abraham Fessehaye Sium, Lina Mohamed, Martha Tesfalul, Filagot Tadesse

**Affiliations:** ^1^ Department of Obstetrics and Gynecology St. Paul's Hospital Millennium Medical College Addis Ababa Ethiopia; ^2^ Department of Internal Medicine St. Paul's Hospital Millennium Medical College Renal Transplant Unit Addis Ababa Ethiopia; ^3^ Department of Obstetrics Gynecology and Reproductive Sciences University of California San Francisco School of Medicine San Francisco California USA

**Keywords:** contraception for renal transplant, contraceptive counselling, renal transplant, renal transplant recipient

## Abstract

**Background:**

Contraceptive counselling and utilization for kidney transplant patients is a vital component of their kidney transplant care. The use of standardized information on contraceptive methods to prevent unplanned post‐transplant pregnancies in Africa in general is less studied. This study aimed to describe contraceptive counselling and uptake among kidney transplant recipients at a kidney transplant centre in Ethiopia.

**Methods:**

A descriptive study on contraceptive counselling and uptake among female Ethiopian kidney transplant recipients was conducted at St. Paul's Hospital Millennium Medical College (Ethiopia) from April 15 to July 15, 2023. Data on women's sociodemographic, renal transplantation and contraceptive counselling and use were collected through interviewing the participants using a structured questionnaire. Data were analyzed on SPSS 23 using simple descriptive analysis. Percentages and frequencies were used to present the results.

**Results:**

A total of 60 participants were included in the final analysis. The mean age of the participants was 33.7 ± 8.4 years. The median duration from the time of renal transplant was 19 months. Most (49/60, 81.7%) of the participants reported that they did not receive family planning counselling on contraceptive methods in the early post‐transplant phase. The rate of contraceptive uptake was 8.3% (5/60) with two patients being copper IUD users, and Implanon, tubal ligation and combined oral contraceptives each utilized by a single kidney transplant patient.

**Conclusion:**

Contraceptive counselling and uptake rates among female kidney transplant recipients in this study were very low, which is consistent with findings from previous studies. Increasing female kidney transplant patients’ awareness on safe and effective contraceptive use through adequate contraceptive counselling is essential.

## Graphical abstract

Contraceptive counselling and uptake rates among female kidney transplant recipients in our study were very low, which is consistent with findings from previous studies. Increasing female kidney transplant patients’ awareness on safe and effective contraceptive through adequate contraceptive counselling is essential.

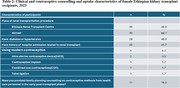

## 1 Introduction

Women awaiting organ transplantation should be advised to use effective contraception, as unplanned pregnancies among such women are common [1]. Although women with end‐stage renal disease are often infertile, their fertility usually returns within few months following renal transplantation [2]. The American Society of Transplantation consensus report recommends waiting at least 1 year after renal transplant before conception and suggests that patients with a recent history of graft rejection or impaired graft function should wait even longer [3], whereas the European guidelines recommend waiting 2 years before conception [4].

Reasons for patients’ choice of contraceptives, physicians’ attitudes toward contraception and medical advice on choice of contraceptive method after renal transplant are scarcely studied [5]. It has been stated that women of reproductive age are at increased risk of unintended pregnancy following the solid organ transplantation (including rental transplantation), and their fertility can return as soon as 1 month after transplant procedure [6]. Most transplant centres encourage women to wait 18–24 months after transplantation before conceiving due to fears of increased transplant complications [7]. A large American cohort study (*n* = 21,814) found that pregnancy within the first year after transplant was associated with increased total and death‐censored graft loss risk and pregnancy in the second year after transplant was associated with increased risk of death‐censored graft loss [8]. There is inadequate literature regarding contraceptive use prevalence and which methods are the most frequently used among these patient population globally. St. Paul's Hospital Millennium Medical College (SPHMMC) is a national kidney transplant centre in Ethiopia. Kidney transplant patients who had transplant in this transplant centre or abroad have their follow‐up at the nephrology clinic within this center. The use of standardized information on contraceptive method and conception to prevent unplanned post‐transplant pregnancies in Ethiopia has not been studied before. This study aims to describe contraceptive counselling and use among female renal transplant recipients of reproductive age in a national kidney transplant in Ethiopia.

## 2 Methods

### 2.1 Study Design, Setting and Sampling

This was a descriptive study of Ethiopian women of reproductive age who had renal transplantation and was conducted at St. Paul's Hospital Millennium Medical College (SPHMMC), Ethiopia, over 3 months (from April 15 to July 15, 2023). SPHMMC is a national kidney transplant centre in Ethiopia. Kidney transplant patients who have had transplant either at SPHMMC or abroad have their routine follow‐up at a transplant clinic within this transplant centre. SPHMMC is also a centre of excellence for family planning (comprehensive abortion care and family planning service), and it is also one of the leading tertiary teaching hospitals in Ethiopia. The renal transplant clinic at this hospital is attended by nephrology specialists and fellows. Participants of this study were women of reproductive age group who had follow‐up at the renal transplant clinic and who volunteered to participate in the study.

### 2.2 Data Collection and Analysis

Data regarding women's sociodemographic, clinical and contraceptive characteristics were collected through interview using a standard questionnaire prepared in English. Data were entered into Epi‐info version 7 and later on transported to SPSS 23 for analysis. Simple descriptive analyses were used to analyze the data. Percentages and frequencies were used to present the results.

### 2.3 Ethics Considerations

Ethical clearance was obtained from the SPHMMC Institutional Review Board. Written informed consent was obtained from the study participants.

## 3 Results

There were 63 renal transplant recipients, out of which 60 were included in the final analysis after three were excluded due to incomplete data. The mean age of the participants was 33.7 ± 8.4 years. Majority (93%, 56/60) of the participants were from urban area. More than half (51.7%, 31/60) of the participants were married (Table 1). Two‐thirds of the participants had their renal transplant abroad, whereas the remaining one‐third had it at the Ethiopia National Kidney Transplant Centre. Close to half (46.7%, 28/60) of the participants were parous. Thirteen (21.7%) women had a history of prior abortion. Among the participants, the median duration since renal transplantation was 19 months.

**TABLE 1:** Sociodemographic and reproductive characteristics of female Ethiopian kidney transplant recipients, 2023.

Characteristics	*n*	%
Age (years)	Mean	33.7 ± 8.4
Residence		
Urban	56	93.3
Rural	4	6.7
Educational level		
High school	27	45.0
College/University	24	40.0
Primary school	7	11.7
Uneducated	2	3.3
Marital status		
Single	26	43.3
Married	31	51.7
Divorced	2	3.3
Widowed	1	1.7
Monthly income (Ethiopian birr)	Mean	3158.3 (±572.13)
Duration since renal transplant (in months)	Median	19 (1–156)
Parity		
Nulliparous	32	53.3
Parous	28	46.7
Had prior abortion?	13	21.7

*Note*: $1 = 55 Ethiopian birr.

Most (49/60, 81.7%) of the participants reported that they did not receive family planning counselling on contraceptive methods from health care personnel in the early post‐transplant phase. The rate of contraceptive uptake was 8.3% (5/60), with two patients being copper IUD users and Implanon, tubal ligation and combined oral contraceptives (COC) each utilized by a single kidney transplant patient (Table 2). Among the IUD users, there was no report of pelvic infection or bothersome uterine bleeding or change in medication or discontinuation of the methods in response to a medical complication. Similarly, there was no history of abnormal uterine bleeding or implant insertion site infection in the Implanon user patient. Likewise, the COC user and the tubal ligation case did not report any concerning complication associated with contraceptive use. Among the contraceptive non‐users, there was one patient who had history of induced abortion for unintended pregnancy.

**TABLE 2:** Clinical and contraceptive counselling and uptake characteristics of female Ethiopian kidney transplant recipients, 2023.

Characteristics of participants	*n*	%
Place of renal transplantation procedure		
Ethiopia Renal Transplant Centre	20	33.3
Abroad	40	66.7
Have diabetes or hypertension	29	48.3
Have history of hospital admission related to renal transplant	25	41.7
Have used modern family planning method after the renal transplant	5	8.4
Intra‐uterine contraceptive device (IUCD)	2	3.3
Contraceptive implant	1	1.7
Combined oral contraceptives (COC)	1	1.7
Tubal ligation	1	1.7
Were you provided family planning counselling on contraceptive methods from health care personnel in the early post‐transplant phase?	11	18.3

## 4 Discussion

Two‐thirds of the participants had their renal transplant abroad, whereas the remaining one‐third had it at the Ethiopia National Kidney Transplant Center. Close to one‐fifth of transplant recipients (18.3%) reported to have received family planning counselling on contraceptive methods from health care personnel in the early post‐transplant phase, and the contraceptive uptake was half of this (8.4%; 1 in 12 women used a contraceptive method).

Currently, little is known about contraceptive use in women with a history of kidney transplants. The few available evidence on this show that contraceptive uptake among these patients is very low. A 2021 cohort study of contraceptive use among women with kidney transplants in the United States, in which 13,150 women who had kidney transplantation between 1 January 2005 and 31 December 2013 aged 15–44 years were analyzed, found a rate of contraceptive use of 9.5% among the participants [9]. The contraceptive uptake found in the study is similar to this report, which is 8.3%. Close to 4 in 5 kidney transplant recipients in our study reported that they did not receive contraceptive counselling from care providers in the early post‐transplant phase, which is higher than reported in a study from Norway, which found more than one‐third (37%) of Norwegian female renal transplant recipients included in the study could not recall having received advice from health care personnel about contraceptive use [5]. Similar to that in our study, a survey of 309 women aged 19–49 years who had received a solid organ transplant, in which 40% were kidney transplant patients, found that only 21.4% reported that a health care provider discussed contraception before transplantation [10]. Another study of 197 kidney transplant recipients concluded that many were not counselled about the need for contraception and did not use any form of birth control, despite the fact that most female kidney transplant recipients were sexually active both before and after transplantation. There were 14 pregnancies in that study, and 13 (92.9%) of them of were unplanned [11]. A recent study from Turkey concluded that the recommendation of contraceptive counselling and utilization for these patients is overlooked with almost one‐third (30.3%) of the patients in the study were not offered contraception [12].

Though very limited evidence on combined oral contraceptives (COC) and transdermal contraceptive patch use among kidney and liver transplant recipients indicated no pregnancies and no overall changes in biochemical measures [13], their interaction with cyclosporine and an increased risk of hypertension preclude them from being a good contraceptive choice for kidney transplant patients [14, 15]. Intra‐uterine devices are convenient contraceptives but have higher failure rate and are associated with an increased incidence of pelvic infection [15]. There is scare evidence on the use of subdermal etonogestrel implant in a woman with a history of transplant. The only available study to date on this compared 24 cases of solid organ transplantation on etonogestrel implant with matching non‐user controls in 1:1 ratio. The study concluded that the etonogestrel contraceptive implant is likely a safe contraceptive option for reproductive‐age women who are solid organ transplant recipients, as the occurrence of graft rejection, pregnancy outcomes, infections and immunosuppressant therapy changes showed no statistically significant difference between either groups [16]. The 2016 US medical eligibility criteria (MEC) put combined hormonal contraceptives as Category 4 for complicated solid organ transplantation and Category 2 if it is uncomplicated one, whereas it classifies IUD as Category 3 for initiation in complicated solid organ transplantation and Category 2 in uncomplicated solid organ transplantation and for continuation in the complicated ones. It also categorizes implant as MEC Category 2 [17]. Two among the contraceptive users in the present study used copper IUD while COC, implant, and tubal ligation were utilized by one user each. Similarly, intrauterine device insertions (2.6%), implant (0.9%) and tubal ligation (0.5%) were utilized in the US study discussed above [9].

This study is among the first to report on contraceptive counselling and use among kidney transplant recipients from a low‐middle income country. It boosts the global inadequate evidence on this topic. Considering the small sample size, low uptake of contraception, and lack of data on the other determinants of contraception uptake, including how many of the participants were sexually active and male partner involvement, we did not perform further analysis to determine factors associated with contraceptive uptake. These are the main limitations of our study. Inability to identify whether the finding of low contraceptive counselling was confounded by the participants recalls bias. Adding qualitative data on providers' attitude and experience would have resulted in stronger results.

## 5 Conclusion

Both contraceptive counselling and uptake rates among female kidney transplant recipients in this study were very low, which is consistent with findings from previous studies. These findings underscore the importance of creating awareness on safe and effective contraceptive use in women with kidney transplant. Optimal contraceptive counselling for such patients should be part of their renal transplantation care, preferably through a multidisciplinary team approach (consisting of a family planning specialist or a gynaecologist and nephrologist).

## Author Contributions

A.F.S. and L.M. developed the concept and design of the project. A.F.S. and L.M contributed to the data collection and data analysis. A.F.S., F.T., L.M. and M.T. contributed manuscript write‐up. All authors checked the manuscript for intellectual contents. The final manuscript is approved for submission by all authors.

## Conflicts of Interest

Authors have no financial or non‐financial competing interests. Abraham Fessehaye Sium is an Editorial Board member of Public Health Challenges and co‐author of this article. He was excluded from editorial decision‐making related to the acceptance of this article for publication in the journal.

## Ethics Statement

Ethical clearance was obtained from St. Paul's Hospital Millennium Medical College IRB. Written informed consent was obtained from study participants.

## Data Availability

The data that support the findings of this study are available from the corresponding author upon reasonable request.
